# A rare metastatic mesenteric malignant PEComa with *TSC2* mutation treated with palliative surgical resection and nab-sirolimus: a case report

**DOI:** 10.1186/s13000-023-01323-x

**Published:** 2023-04-11

**Authors:** Luke Meredith, Timothy Chao, Avinoam Nevler, Atrayee Basu Mallick, Rajan K. Singla, Peter A. McCue, Wilbur B. Bowne, Wei Jiang

**Affiliations:** 1 Department of Surgery, Sidney Kimmel Cancer Center, Thomas Jefferson University Hospital, Philadelphia, PA 19107 USA; 2 Department of Pathology and Genomic Medicine, Sidney Kimmel Cancer Center, Thomas Jefferson University Hospital, Philadelphia, PA 19107 USA; 3 Department of Medical Oncology, Sidney Kimmel Cancer Center, Thomas Jefferson University Hospital, Philadelphia, PA 19107 USA

**Keywords:** Malignant PEComa, Nab-sirolimus, mTOR, TSC2, TP53

## Abstract

**Background:**

Malignant perivascular epithelioid cell tumors (PEComas) are exceedingly rare malignant mesenchymal neoplasms with characteristic morphological and immunohistochemical (IHC) patterns. However, some malignant PEComas are poorly differentiated with atypical histopathological features, making a definitive diagnosis difficult. PEComas are most commonly found in females and often show either *TSC1* or *TSC2* alterations, which result in the activation of the mTOR pathway, or *TFE3* fusions. Given these molecular characteristics, mTOR inhibitors have recently been approved by the FDA in the treatment of malignant PEComas, particularly in those with *TSC1/2* alterations. Therefore, molecular analyses may be helpful for both the diagnostic workup of and predicting response to mTOR inhibitors in cases of malignant PEComas.

**Case presentation:**

Here, we report a case of an aggressive, 23 cm mesenteric malignant PEComa with multiple peritoneal metastases in a young male patient. Pathological examination of the initial biopsy showed a malignant epithelioid neoplasm with high-grade morphology and atypical immunoprofile, which precluded a definitive diagnosis. Because of the patient’s excessive transfusion requirements due to intra-tumoral hemorrhage, a palliative R2 resection was performed. Histopathological examination of the tumor revealed focal immunoreactivity for Melan-A, HMB-45, desmin, and CD117. Although a diagnosis of malignant PEComa was favored, other entities such as epithelioid gastrointestinal stromal tumor (GIST) or melanoma could not be definitively ruled out. Given the favored diagnosis, the patient was started on sirolimus, an mTOR inhibitor, rather than chemotherapy. Molecular analyses were performed and the tumor was found to harbor mutations in *TP53* and *TSC2*, supporting a definitive diagnosis of malignant PEComa. The patient was then switched to nab-sirolimus, with initial stabilization of the disease.

**Conclusions:**

This report details a multidisciplinary approach for the diagnosis and management of a highly aggressive, metastatic malignant PEComa in a young male patient. The basis for the treatment of malignant PEComas with the recently FDA-approved mTOR inhibitor, nab-sirolimus, is also reviewed. In summary, this case highlights the importance of molecular analysis, particularly *TSC1/2* alterations, for both the definitive diagnosis of malignant PEComas and predicting their response to nab-sirolimus.

**Supplementary Information:**

The online version contains supplementary material available at 10.1186/s13000-023-01323-x.

## Background

Perivascular epithelioid cell tumors (PEComas) are rare mesenchymal tumors with characteristic perivascular distribution of epithelioid cells which are immunoreactive of both smooth muscle and melanocytic markers. Definitive diagnosis of PEComas relies largely on histological examination with characteristic morphological and immunohistochemical (IHC) findings. However, diagnosing PEComas may be difficult in cases with poor differentiation or atypical immunophenotypes. Genetic studies have recently demonstrated that most PEComas harbor either TSC1 or TSC2 alterations, both of which result in the activation of the mammalian target of rapamycin (mTOR) signaling pathway and tumor proliferation [1–3]. In addition, a small subset harbors TFE3 fusions [4]. Therefore, molecular studies should be considered especially in cases of high-grade epithelioid mesenchymal tumors to help support a diagnosis of PEComas.

While the majority of PEComas are indolent, some can display a malignant clinical course. Malignant PEComas are exceedingly rare with an incidence of < 1/1,000,000 and their management is actively evolving [2, 5]. Radical surgical resection remains the primary treatment for PEComas because they tend not to respond to radiation and chemotherapy [6]. For locally advanced or metastatic PEComas, previous standard of care involves palliative chemotherapy containing doxorubicin, gemcitabine, or ifosfamide, with only short-term responses reported in literature [6]. Indeed, mTOR inhibitors have recently demonstrated significant efficacy and have been FDA-approved as a treatment for malignant PEComas [7, 8]. In this work, we present a multidisciplinary approach for the diagnosis and treatment of an aggressive mesenteric malignant PEComa in a young male patient, highlighting the importance of molecular analysis.

## Case presentation

A 26-year-old male with a history of asthma initially presented to the emergency department with two weeks of progressively worsening constipation, early satiety, and a twenty-pound unintentional weight loss over less than 3 months. On presentation, patient was found to be febrile and tachycardic. Further workup showed severe iron deficiency anemia with hemoglobin below 5 gm/dl. Computed tomography (CT) imaging demonstrated a 20 × 10 × 15 cm abdominopelvic mass and extensive peritoneal disease (Fig. 2A). Ultrasound-guided core-needle biopsy was performed on the main mass, which showed a malignant epithelioid neoplasm. IHC studies demonstrated that the tumor cells were patchily positive for CD117, Melan-A and desmin, but negative for DOG1. Additional markers for hepatocellular carcinoma, lymphoma, and germ cell neoplasia proved negative. A definitive diagnosis could not be made. Given its minimal CD117 expression, a variant of gastrointestinal stromal tumor (GIST), such as succinate dehydrogenase (SDH)-deficient GIST, was also considered.

The patient was discharged but had to return one week later to the emergency department due to symptoms related to worsening anemia. Abdominal CT imaging at the time showed that the intra-peritoneal mass had grown to 23 × 10 × 17 cm, which substantially increased in size compared to initial presentation one week earlier. The mass now affected the bowel and there was bilateral hydronephrosis. Imaging also noted the tumor was highly vascular with extensive collateralization. A second ultrasound-guided core-needle biopsy was performed. Light microscopy showed a high-grade malignant epithelioid neoplasm, similar to the previous biopsy. Additional staining with Cam 5.2, S100, Sox10, MyoD1, DOG1, ERG, and ALK1were negative. INI1 staining is retained. Again, the lack of lineage-specific immunophenotyping precluded a definitive diagnosis.

The patient was transferred to a tertiary care center for further evaluation. His fever, tachycardia, and leukocytosis were attributed to a systemic inflammatory response to intra-tumoral necrosis and hemorrhage. Unfortunately, persistent intra-tumoral hemorrhage and anemia necessitated 8 units of red cell transfusions over a span of 3 weeks. Due to transfusion requirements, poor nutritional status, rapid clinical deterioration, and the need for additional diagnostic material, palliative surgical resection was advised.

The patient underwent selective gel foam embolization of the omental artery and tumoral arteries from the right gastroepiploic artery. Following the embolization procedure, palliative omentectomy and tumor debulking were performed for symptom control. At surgery, the tumor appeared to arise from the transverse colon mesentery with multiple peritoneal nodules and malignant adhesions to the bowel and bladder wall. The tumor was noted to be friable and highly vascular. The resected portion of the tumor was sent to pathology for evaluation.

**Fig. 1:**
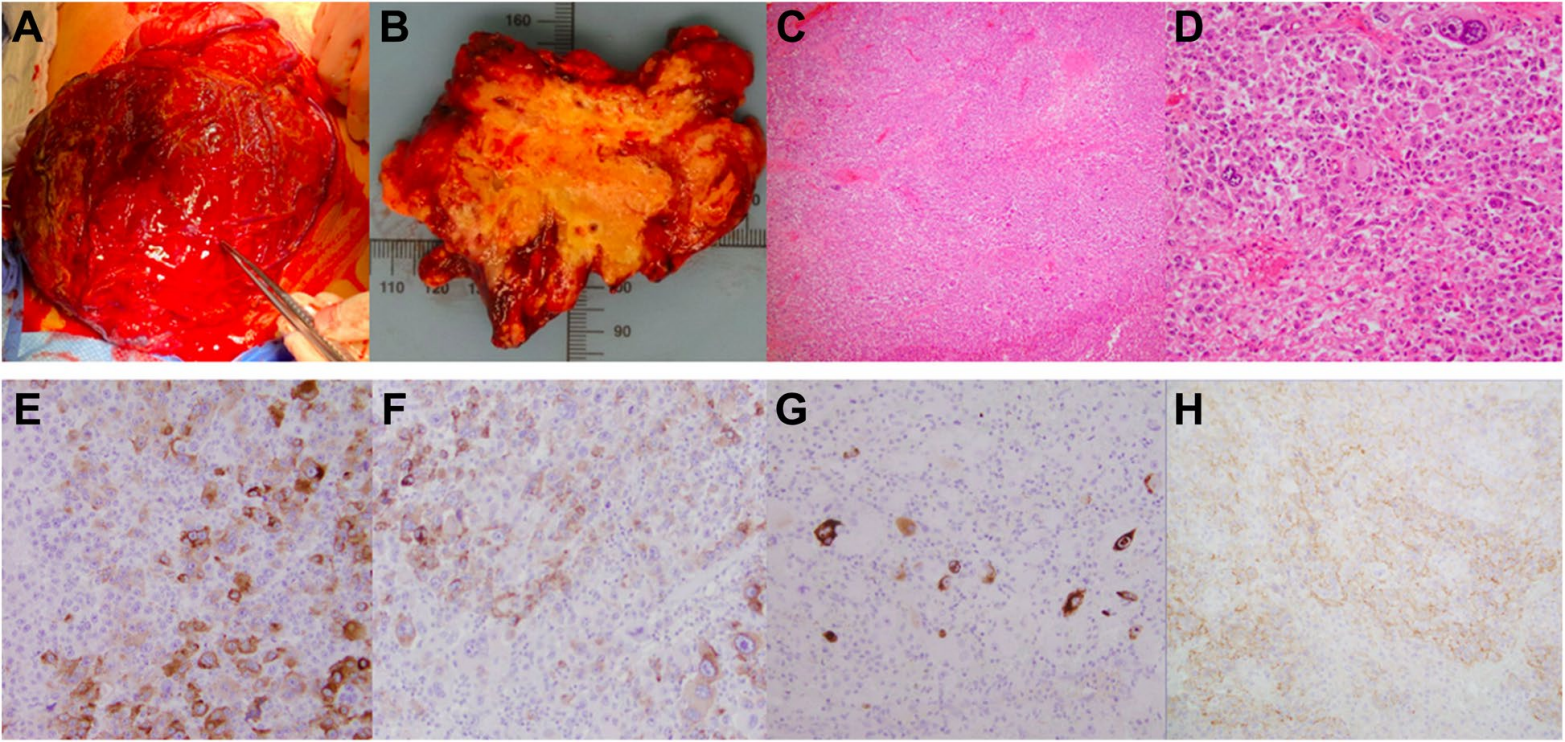
Gross, light microscopic, and IHC findings. **A** Intra-operative gross photo of the tumor. **B** Representative section of tumor consisting of fleshy surfaces with extensive necrosis and hemorrhage. **H**&**E** stain showing sheets of malignant epithelioid cells with granular eosinophilic cytoplasm, marked nuclear atypia, and prominent nucleoli, 4x (**C**) and 20x (**D**). Tumor cells show patchy positive staining for Melan-A (**E**), HMB-45 (**F**), desmin (**G**), and CD117 (**H**)

Gross examination of the resected tumor (Fig. 1A) and serial sections (Fig. 1B) showed a tan-red, fleshy mass, with significant areas of necrosis and hemorrhage. Histologic examination of the specimen demonstrated extensive necrosis and solid, sheet-like proliferation of large epithelioid cells with granular eosinophilic cytoplasm. There was also marked nuclear atypia, with prominent nucleoli, and nuclear pleomorphism (Fig. 1C and D). Brisk mitotic activity (> 100 per 50 HPFs) with atypical mitoses were observed. Additional IHC studies were completed in another attempt to make a definitive diagnosis for this tumor. The malignant cells showed focal patchy positivity for Melan-A, HMB-45, and desmin (Fig. 1E, F, and G, respectively). There was also again focal weak staining for CD117 (Fig. 1H). The tumor cells were negative for AE1/AE3, Cam5.2, Sox10, S100, CD3, CD20, CD79a, CD30, CD138, CD56, DOG1, SMA, MyoD1, myogenin, chromogranin, synaptophysin, neurofilament, GFAP, ALK1, OCT3/4, inhibin, WT-1, calretinin, ERG, and GATA3. In addition, the tumor cells showed retained INI-1, BRG1/SMARCA4, and SDHB stainings. These findings were compatible with a high-grade sarcoma with epithelioid morphology. In light of these findings, a diagnosis of malignant epithelioid PEComa was favored. Other neoplasms with epithelioid morphology, including clear cell sarcoma of the GI tract, gastrointestinal neuroectodermal tumor (GNET), melanotic schwannoma, epithelioid sarcoma, germ cell tumor, anaplastic large cell lymphoma, are considered much less likely based on the IHC profiles. Epithelioid GIST and malignant melanoma were unable to be ruled out at that time.

The patient’s post-operative course was complicated by ileus, requiring nasogastric tube placement and total parenteral nutrition. Within 7 days of the debulking surgery, the patient was started empirically on oral sirolimus given that a diagnosis of malignant PEComa was favored with gross residual disease and a rapidly growing tumor. The plan was to reassess the definitive systemic therapy after molecular results were available. On post-operative day 9, CT scan showed significant jejunal obstruction caused by rapid post-operative disease progression from residual intra-abdominal disease. An endoscopic lumen apposing metal stent (LAMS) was placed to bypass the obstructed bowel and was successful. The patient was then able to tolerate a normal diet.

An in-house hotspot gene mutational panel for 30 selected cancer-related genes and a RNA-based next generation sequencing sarcoma/solid tumor gene fusion panel of 99 oncogenic genes were performed to support the favored diagnosis and help rule out other entities. The gene mutational panel demonstrated a pathologic *TP53*-p.R337C missense mutation, with an allele frequency of 52.5% (Supplemental Fig. 1A). Notably, lack of *BRAF, NRAS, c-KIT,* and *PDGFRA* hotspot mutations in conjunction with the histological findings above confirmed that melanoma and conventional GIST were unlikely. The gene fusion panel was negative for *EWSR1, CIC, BCOR,* and *TFE3* translocations. Formalin-fixed paraffin-embedded tissue was sent for next generation whole exome sequencing (Caris Life Sciences, Irving, TX). In addition to the *TP53* mutation observed by the in-house gene mutational panel, the results from whole exome sequencing showed a pathologic *TSC2-*p.K1165fs mutation with an allele frequency of 58% (Supplemental Fig. 1B). Considering the clinical presentation, light microscopy, immunophenotyping, and molecular alterations, a definitive diagnosis of malignant epithelioid PEComa was rendered.

**Fig. 2:**
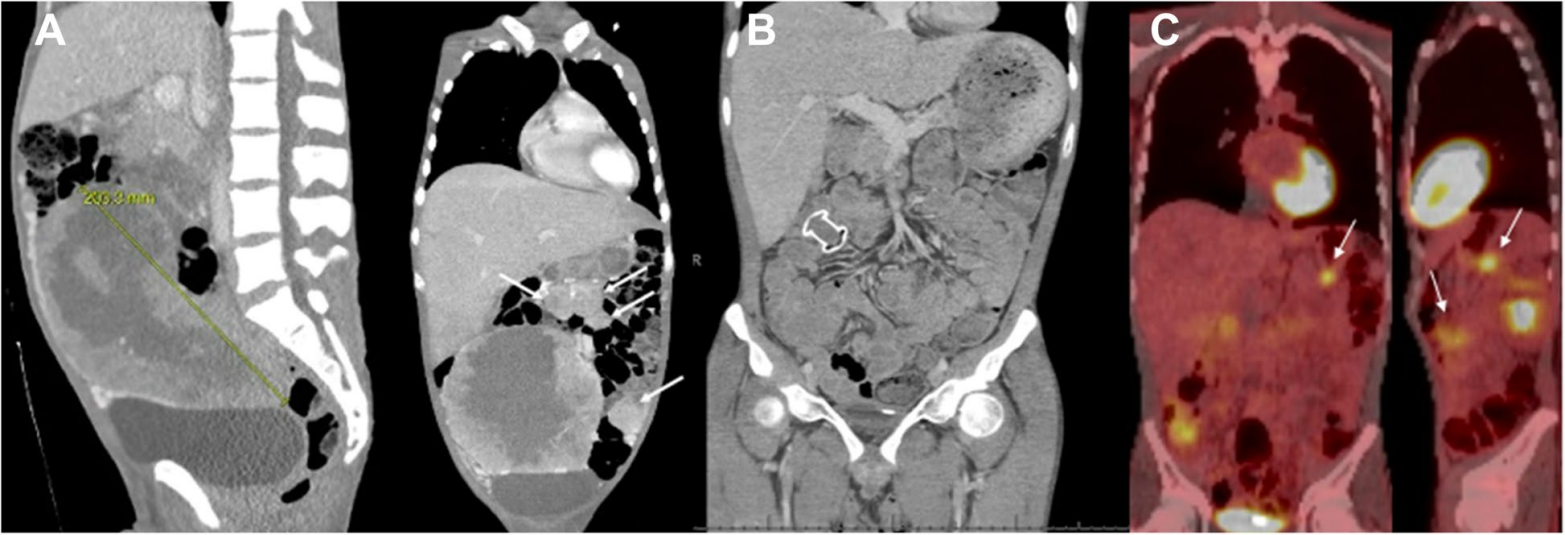
Radiographic image findings. **A** Sagittal and coronal CT abdominal images on initial presentation showing a large abdominopelvic mass. White arrows in coronal view highlight metastatic disease in the abdomen. **B** Coronal CT abdominal post-surgical interventions showing absence of the main mass and the LAMS stent. **C** Coronal and sagittal PET CT images one month post initiation of nab-sirolimus showing stable residual disease in the abdomen as highlighted by the white arrows

With this definitive diagnosis, the patient was switched to IV nab-sirolimus given poor reliance on oral drug absorption due to bypass of portions of his gastro-intestinal tract via the LAMS procedure. PET CT imaging one month after starting nab-sirolimus demonstrated stable residual abdominal disease (Fig. 2C). However, PET CT demonstrated progression of disease after 2 cycles of nab-sirolimus with 2 treatment breaks. Patient was subsequently switched to gemcitabine and docetaxel. After 2 cycles of the chemotherapy regimen, sirolimus was again added given the tumor’s prior response and the patient’s clinical status.

## Discussion

PEComas are defined by the World Health Organization (WHO) as mesenchymal neoplasms composed of perivascular epithelioid cells that are closely associated with blood vessel walls. These tumors co-express melanocytic and smooth muscle markers [9]. The PEComa “family” includes entities such as angiomyolipoma, clear cell “sugar” tumor of the lung, lymphangioleiomyomatosis, primary extrapulmonary sugar tumor, clear cell myomelanocytic tumor of the falciform ligament/ligamentum teres, and abdominopelvic sarcoma of perivascular epithelioid cells [9, 10]. PEComas usually present in young to middle-aged adults (mean of 45 years) and occur more frequently in females, with a male to female ratio of 1:5 [11–13]. The majority of cases are sporadic, but a small subset is associated with tuberous sclerosis [14, 15]. The tumors typically consist of epithelioid or spindled cells with granular eosinophilic to clear cytoplasm that are arranged in a nested, trabecular, or sheet-like pattern. Melanoma-like nucleoli and intranuclear pseudo-inclusions may be present.

Although most cases follow a benign clinical course, a small subset of PEComas do behave aggressively [12, 13, 15, 16]. Multivariate analyses show that malignant potential of PEComas can be predicted based on the following 6 high-risk features: size > 5 cm, infiltrative growth, high grade nuclear atypia, mitoses > 1 per 50 high power fields (HPF), necrosis, and lympho-vascular invasion (LVI). Non-gynecologic PEComas are classified as malignant if they possess 2 or more of these high-risk features. Tumors with uncertain malignant potential contain nuclear atypia or have size ≥ 5 cm. Otherwise; benign tumors show no high-risk features (Table 1) [12]. Multiple malignancy criteria for gynecologic PEComas have been proposed, which generally include tumors with 3–4 or more high-risk features, excluding the infiltrative pattern [15, 17].

**Table 1:** Classification of malignant potential for non-gynecologic PEComas (adapted from [12])

High risk features	Risk category
(1) Size > 5 cm (2) Infiltrative growth pattern (3) High nuclear grade and cellularity (4) Mitotic Rate > 1/50 HPF (5) Necrosis (6) Vascular invasion	• “Benign”: < 2 high risk features and size < 5 cm • “Uncertain malignant potential”: Size ≥ 5 cm with no other high risk features OR nuclear pleomorphism/multinucleated giant cells only • “Malignant”: ≥ 2 high risk features

Two major molecular subgroups have recently been identified for PEComas: those with *TSC1* or *TSC2* alterations and those with *TFE3* fusions [3, 4, 18]. Interestingly, Schmeister et al. recently reported a case of aggressive PEComa with simultaneous TSC1 mutation and TFE3 inversion, which suggests that these two pathways may not be mutually exclusive [19]. Loss of heterozygosity in the *TSC2* locus, such as deletion of 16p or *TSC2* mutations, resulting in loss of function of the tuberin protein are commonly found in PEComas [18]. Tuberin forms a heterodimer with the protein product of *TSC1*, hamartin, which functions as a tumor suppressor by inhibiting the mTOR signaling pathway. Loss of tuberin function or hamartin results in the uncontrolled activation of mTOR signaling and potential tumorigenesis. Concurrent *TP53* mutations are found in up to 63% of *TSC2*-mutated PEComas, the significance of which remains to be further delineated [3]. A second subset of PEComas characterized by their prominent alveolar growth pattern and lack of smooth muscle marker expression harbor *TFE3* rearrangements [3, 4]. These *TFE3*-rearranged PEComas tend to occur in younger patients and display strong nuclear TFE3 expression. TFE3 is a transcription factor that forms a homodimer or heterodimer commonly with MiT-related proteins, such as TFEB, to promote cellular growth and proliferation [20]. Furthermore, renal cell carcinoma cells in vitro with TFE3 fusions are capable of growth independent of mTOR signaling [21].

Our patient’s malignant PEComa is unusual in many aspects. Firstly, it occurred in a young 26- year-old male whereas the majority of PEComas (> 80% reported in the literature) are found in females [12]. There was no family history of tuberous sclerosis, and the patient did not have any other clinical manifestations of the syndrome. However, given the young age, the patient was referred to genetic counseling for assessment for further germline testing. Secondly, mesenteric PEComas are exceedingly rare. To date, only 14 cases are reported in the literature. Interestingly, most of these cases exhibited an epithelioid morphology and had favorable outcomes [12, 22–31]. Thirdly, our patient’s tumor presented as an unusually aggressive disease with rapid growth and multiple metastases, fulfilling all 6 high-risk histologic features for malignant PEComas. Lastly, although suggestive of the diagnosis, this tumor’s immunophenotype was not entirely specific for PEComa. Only patchy focal expression of a single smooth muscle marker (desmin) and 2 melanocytic markers (HMB45 and Melan-A) were observed. The initial differential diagnosis list is broad, including epithelioid PEComa, epithelioid GIST, malignant melanoma, clear cell carcinoma of the GI tract, GNET, melanocytic schwannoma, epithelioid sarcoma, epithelioid inflammatory myofibroblastic tumor, germ cell tumor, and others were also on the differential. Indeed, a study found that nearly 33% of epithelioid GISTs show at least focal staining of Melan-A [32] Others have reported expression of smooth muscle markers, including desmin, in a subset of GISTs [33, 34]. Furthermore, some mucosal melanomas can also stain for desmin [35, 36]. These two entities remain remote diagnostic possibilities, with other entities ruled out based on IHC profiles. Our present case highlights the real-world difficulty in making a definitive diagnosis of malignant PEComa based solely only on light microscopy and IHC.

The molecular findings in this case were instrumental in confirming the diagnosis. Gene sequencing identified pathologic *TP53-*p.R337C and *TSC2-*p.K1165fs mutations, and showed no *BRAF, NRAS, CKIT,* and *PDGFRA* hotspot mutations. These results rendered melanoma and conventional GIST highly unlikely and confirmed the diagnosis of a PEComa. Lack of TFE3 nuclear immunostaining and *TFE3* fusions by our in-house gene fusion panel further ruled out a *TFE3*-rearranged PEComa. Although frame-shift mutations in *TSC2* are common in multiple disease states, the pathologic *TSC2*-p.K1165fs frame-shift mutation involving exon 30 seen in this tumor has not been previously reported in literature.

Detection of a *TSC2* mutation not only confirmed the diagnosis, but also suggested that our patient may benefit from the recently FDA-approved mTOR inhibitor, nanoparticle albumin-bound (nab) sirolimus. Intravenous nab-sirolimus was shown in pre-clinical studies to have higher intra-tumoral drug accumulation, mTOR suppression, and tumor growth inhibition compared to its oral counterparts, such as sirolimus and everolimus [37, 38]. The recent prospective, phase II AMPECT clinical trial, which led to the FDA approval of nab sirolimus for malignant PEComa, reported that nab-sirolimus provided a 39% overall response rate in malignant PEComas [8]. Interestingly, the response rate was 89% in PEComas with a *TSC2* mutation, whereas it was 13% without. Indeed, treatment of our patient’s unusually aggressive malignant PEComa with nab-sirolimus showed initial objective evidence of disease control by cross-sectional/nuclear imaging [39].

Limitations of this case report include the retrospective nature of this review, short duration of follow-up and requirement for more patients to validate the diagnostic and treatment strategy presented herein. Although generalities are limited in a case report, our case highlights the importance of molecular tumor profiling in conjunction with light microscopy and IHC findings for the definitive diagnosis and identification of novel actionable targets in malignant PEComas.

## Supplementary Information

**Additional file 1: Supplemental Figure 1.** Molecular findings. (A) Results from in-house hotspot gene mutational panel reporting a TP53-pR337C mutation. (B) Summary of the Caris Molecular Intelligence Tumor Profiling report highlighting the TSC2 p.K1165fs mutation.

## Data Availability

All data and materials generated or analyzed during this study are included in the article.

## Abbreviations

CT: Computerized tomography
FDA: Food and Drug Administration
GIST: Gastrointestinal stromal tumor
H&E: Hematoxylin and eosin
HPF: High-power field
IHC: Immunohistochemistry
LAMS: Lumen apposing metal stent
LVI: Lymphovascular invasion
mTOR: Mammalian target of rapamycin
nab-: Nanoparticle albumin-bound-
PEComa: Perivascular epithelioid cell tumors
PET: Positron emission tomography
WHO: World Health Organization
GNET: Gastrointestinal neuroectodermal tumor
