# Supplementary material for: A rare metastatic mesenteric malignant PEComa with TSC2 mutation treated with palliative surgical resection and nab-sirolimus: a case report
Source: Diagn Pathol. 2023 Apr 11;18:45. doi: 10.1186/s13000-023-01323-x (PMC10088294; doi:10.1186/s13000-023-01323-x)
Supplement: Supplementary file 1 — Additional file 1: Supplemental Figure 1. Molecular findings. (A) Results from in-house hotspot gene mutational panel reporting a TP53-pR337C mutation. (B) Summary of the Caris Molecular Intelligence Tumor Profiling report highlighting the TSC2 p.K1165fs mutation. [file 13000_2023_1323_MOESM1_ESM.docx]

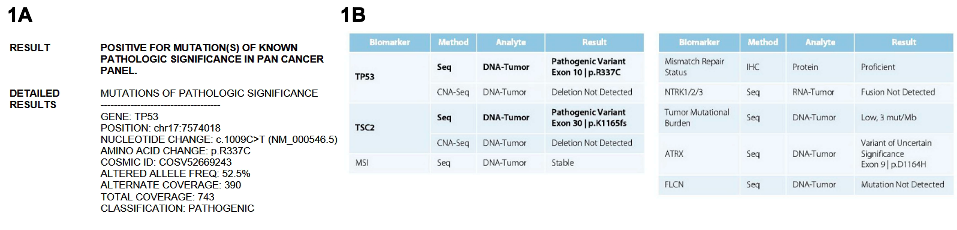


Supplemental Figure 1. Molecular findings. (A) Results from in-house hotspot gene mutational panel reporting a TP53-pR337C mutation. (B) Summary of the Caris Molecular Intelligence Tumor Profiling report highlighting the TSC2 p.K1165fs mutation.
